# 

**Published:** 1896-12-19

**Authors:** 


					192 THE HOSPITAL. Dec. ]9, 1896.
EARLY EDITION.
Notices of Births, Deaths, and Marriages are inserted in this
? column at a oharge of 2s. 6d. for an announcement not exceeding
fonr lines.
NOTICE TO CORRESPONDENTS.
? All MS., Letters, Books for Review, and other matters intended for
the Editor should be addressed The Editor, " The Hospital," The
Hospital Building, 28 & 29, Southampton Street, Strand, W.O.
The Editor cannot undertake to return rejected MS., even when
accompanied by stamped directed envelope.
Uotice to Advertisers and Subscribers.
AH Advertisements, Orders for Copies of The Hospital, and Business
Communications should be addressed to THE MANAGER,
(Not to The Editor), The Hospital, The Hospital Building, 28 & 29,
Southampton Street, Strand, London, W.O.
The Cost of Subscription, post free, i3 as follows:?Per week,_ 2$d.;
per quarter, 2s. 8d.; per half-year, 5s. 3d.; per annum, 10s. 6d. Foreign :?
Per annum, 15s. 2d.; payable, in all cases, in advance.
NOTICE.?1Vol. XX. of "The Hospital" (April to Sep-
tember, 1896), in handsome cloth binding, is now
on sale at the Office of this Journal, price 7s. 6d.
Cases for binding the Numbers contained in this
Volnme Is. 6d. Index to Vol. XX., 2$d., post free.
The Monthly Fart for December is now ready, Price 6d.f
by post 8d.
IMPORTANT NOTICE.
On account of the Christmas Holidays, we beg to
announce that we shall sro to press with our issue of the
2?th INSTANT on TUESDAY NIGHT, the 2ind INST.
Advertisements inteuiei for insertion in thisissue should
therefore reach us BY NOON on TUESDAY, the a* nd inst.
Scraps and Gleanings.
In a recent action of appeal against a decision by Mr. Justice Keke-
wich, which restrained a certain company from passing off meat extract
sold by them as meat extract sold by Liebigf's Extract of Meat Company
(Limited), the appeal was dismissed with costs.
The Treasurer of the Liverpool Northern Hospital has received ?500
from the late Mr. Edgar S. Holland towards the new hospital syecial
account, and a donation of ?200 from Mr. Walter Holland for the
ambulance fund of the hospital.
On Friday, the 18th inst., the Princess Christian will visit the North
London Hospital for Consumption for the purpose of unveiling the
memorial to the late Mrs. Rundle Charles.
At the request of the Prince Regent of Bavaria, Professor Max von
Pettenkofer, the eminent scientist and director of the scientific depart-
ment of Liebig's Extract of Meat Company (Limited), has consented to
fill the chair at the Academy of Science at Bavaria, and to act as Keeper
of the State Scientific Collection for a further term of three years
206 THE HOSPITAL. Dec. 19 1896.
The Student of Medicine.
APPOINTMENTS.
C. Addison, M.D.Lond ,F.R.C.S.Eng., appointed Arthur Jack-
son Professor of Anatomy in the Sheffield School of Medicine.
R. A. Beaver, M.D.Edin., L.E.C P., M.R.C.S., appointed
Medical Officer for the Hinton St. Mary District of the
Sturminster Union and Deputy Coroner for North Dorset. J. S.
Boden, M.R.C.S.Eng, L.R O.P.Lond., appointed Assistant
Medical Superintendent to the Higbgate Infirmary of the St.
Pancras Union. J. S. Boyd, L.E.C P., L.R.C.S.Edin., appointed
District Medical Officer of the "West Ham Union. G. Dalton,
L.S.A.Lond., appointed Surgeon to. cable ship "Electra" of
Eastern Telegraph Service. Dr. Davidson, appointed Medical
Officer of the Uringley District of the Retford Union. W. J.Forbes,
M.B., appointed Medical Officer for the Scriven District of the
Knaresborough Union. L. G. Fraser, B.S , M.B., appointed
Honorary Surgeon to the Tynemouth Infirmary. P. Frazer,
M.D.Edin., appointed Medical Officer of Health to the Uolwyn
Bay and Colwyn Urban District Council. Dr Fulton, appointed
Medical Officer of Health to the Parish of Stevenston. J. S.
Hinnell, B.A.Camb., M.D., M.R.C.S., appointed Medical Officer
for the No. 6 District of the Thingoe Union. R. Jardine,
M.D.Edin., M.R.C.S.Eng., F.F.P. and S.Glasg., appointed
Physician to the Glasgow Maternity Hospital. W. S. Lang-
worthy, M.R.C.S., L.R C.P.Lond., appointed Medical Officer
for the No. 4 District of the Plympton St. Mary
Union. A. McWilliam, M.A., M.B.. C M., appointed Resident
Medical Superintendent, Heigham Hall Asylum, Norwich.
G. E. A. Morshead, M.D.Brux., L.R.C.P.Lond., M.R.C S.Eng.,
appointed Medical Officer for the Guildford District of the
Guildford Union. G.Newman, M.D.Edin.; appointed Medical
Officer for the Charterhouse Parish of the Holborn Union.
H. W. Newton, L.R.C P.Lond., M.R.C.S.Eng., appointed
Medical Officer of Health to the Borough of Chelmsford and a
Joint Medical Officer to the Chelmsford Infirmary and Dispen-
sary. E. P. Normian, B.A., M.D.T.C.D., appointed Medical
Officer of the Bosmers and Claydon Union .Workhouse and
Coddenham District. E. J. P. Olive, M.A., M.D.Cantab.,
F.R.C.S.Eng., appointed an Honorary Surgeon to theWarne-
ford Hospital, Leamington. C. E. Potter, M.B., C.M.Edin.,
appointed Medical Officer of Health to the Asliborne Rural
District Council. J. K. Raymond, M.B., C.M., appointed Resident
Medical Officer to the York Dispensary. A. L. Salmon, M.R C.S.,
L.S.A., appointed Medical Officer for the St. Clement District
and the Workhouse of the Truro Union. W. J. E Sumpter,
M.E.G.S.Eng., L.R C.P.Lon., appointed Medical Officer for the
Sheringham District of the Erpingham Union. E. C. Thomas,
M.B., C M.Edin., appointed Medical Officer of Health to the-
Lampeter (Cardigan) Rural District Council. E. Thomas, M.B.,.
L.R.C.P.Lond., M.R.C.S.Eng , appointed Clinical Assistant to-
the Highgate Infirmary of the St. Pancras Union. H. E..
Thompson, M.B.Lond., M R.C S.Eng., L.R.C.P.Lond., ap-
pointed Junior House Surgeon of the Blackburn and East Lan-
cashire Infirmary. R. H. Trotter, M B , B Ch.Vict., appointed
Medical Officer of Health to the Holme, Holmfirth, Honley, audi
Netherthong Urban District Council. S. S. Whielis, M.D.f
B.S.Durh., appointed an Assistant Surgeon to the Flemming
Memorial Hospital for Sick Children, Newcastle-on-Tyne. ?
Society of Apothecaries of London.
Pass List, November, 1896.
The following candidates passed in :?
Surgery.?J. H. Chambers, Manchester; H. Cross, Sheffield and!
London; A. G. Everard, Charing- Cro3S; S. H. Greene, Charing- Cross ;
A. J. A. Lennane, University College : E. A. McAnally, St. George's; W.
Mawer, St. Bartholomew's; J. P. Race, Manchester; W. F. Reckitt,
Guy's; A. W. Robertson, St. Bartholomew's; G. E. H. Sargent,London;
J. A. Williams, McGill.
Medicine, Forensic Medicine, and Midwifery.?H. Cross, Sheffield
and London; A. J. A. Lennane, University College; A. W. Oxford,
Charing Cross.
Medicine and Forensic Medicine.?M. Sharp, Royal Free.
Medicine and Midwifery.?G. R. B.Gaudoin, Madras; W. A. Pierce,
Liverpool; J. P. Race, Manchester.
Medicine.?T. S. Biggs, Guy's ; G. B. Brown, Guy's; G.C.Jackson,
Cambridge and St. Mary's; A. "VV. Shea, Sheffield and Dublin.
Midwifery.?J. B. Cautley, St. Bartholomew's; A. D. Davies,
London.
The diploma of the Society was granted to the following candidates t
Messrs. Biggs, Brown, Cross, Davies, Everard, Gaadoin, Greene, Jackson,
Lennane, McAnally, Pierce, and Williams.
BIRMINGHAM GENERAL HOSPITAL
FOUNDED 1772.
SUPPORTED BY VOLUNTARY CONTRIBUTIONS,
?4,500
THE ANNUAL EXPEND!
TUBE EXCEEDS
( INCOME FROM INVESTMENTS
|?16,600
?5,000
ANNUAL SUBSCRIPTIONS
270 Beds. Daily average occupied, 230.
During the year 1895 there were admitted 4,461 In-patients and 52,608 Oat-patients (a total of 57,069
cases), of which 4,147 In and 36,786 Oat Patients were admitted without Ticket.
A NEW BUILDING- is now being erected to contain 340 Beds. FUNDS ARE URGENTLY
NEEDED FOR ITS COMPLETION.
The JafFray Branch at Gravelly Hill (56 Beds) receives the more chronic cases from
the "Wards, and so enables a larger number to be admitted to the parent Institution.
Separate Subscription List of ?600. | Expenditure over ?3,000.
NEW AND INCREASED ANNUAL SUBSCRIPTIONS ARE URGENTLY NEEDED,
and will be thankfully received by
HOWARD J. COLLINS, House Governor.
12 TBE HOSPITAL.?CHRISTMAS APPEAL SUPPLEMENT. d?c. 19,183S.
CHELSEA HOSPITAL ? WOMEN,
FULHAM ROAD, S.W.
Pafrwi?H.R.H. THE PRINCESS OP WALES.
Chairman?The LORD GLENESK. Treasurer? HENRY E. WRIGHT, Esq.
Bankers?LONDON AND COUNTY, Sussex Place, S.W.
52 Beds for the cure and relief of diseases peculiar to the female sex.
Has no endowment or invested property.
Its Convalescent Home, with 22 Beds at St. Leonard's-on-Sea, is
not limited to the admission of Hospital patients only.
New Annual Subscriptions and Donations are much needed
by both Institutions.
HERBERT H. JENNINGS, Secretary.
32 THE HOSPITAL.?CHRISTMAS APPEAL SUPPLEMENT. Dec. 19, i89s.
ROYAL LONDON OPHTHALMIC HOSPITAL,
MOORFIELDS,
FUNDS MUCH NEEDED.
Total Ordinary Income ... ... ...   ?"4,068 5 7
Total Ordinary Expenditure    7>034 7 7
Patients   28,474
Attendances  131,450
Annual SUBSCRIPTIONS and DONATIONS earnestly solicited.
BiDbtrt : Metsrs. Williams, Deacon, and Co., 20, Birchin Lane, E.G.
ROBERT J. NEWSTEAD, Sfcretary.

				

